# Anti-COVID-19 drug screening: Frontier concepts and core technologies

**DOI:** 10.1186/s13020-020-00393-z

**Published:** 2020-10-28

**Authors:** Hua Luo, Mingming Zhao, Dechao Tan, Chang Liu, Lin Yang, Ling Qiu, Yan Gao, Hua Yu

**Affiliations:** Institute of Chinese Medical Sciences, State Key Laboratory of Quality Research in Chinese Medicine, University of Macau, Room 8008, Building N22, Avenida da Universidade, Taipa, Macao SAR China

**Keywords:** COVID-19, Disease model, Core technology, Traditional Chinese medicine

## Abstract

The outbreak of COVID-19 has recently evolved into a global pandemic. Up to July 2020, almost every country has confirmed COVID-19 cases reported worldwide. Many leading experts have predicted that the epidemic will persist for relatively a long period of time. Thus far, there have been no remedies proven effective against the disease. As the nation where COVID-19 broke out first, China has adopted a combination of traditional Chinese medicine and western medicine to fight against the disease, and has achieved significant clinical result. Up to now, the COVID-19 pandemic has been effectively controlled in China. However, the rest of the world (except for a limited number of countries and regions) is still in deep water. This paper thoroughly summarizes interdisciplinary notions and techniques, including disease model, biochip, network pharmacology, and molecular docking technology, etc., providing a reference for researchers in the screening of drugs for COVID-19 prevention and treatment. These methodologies may facilitate researchers to screen out more potential drugs for treating COVID-19 pneumonia and to tackle this global crisis.

## Introduction

COVID-19 is an acute respiratory infection caused by a novel coronavirus (SARS-CoV-2). The main symptoms of this disease include fever, dry cough, and fatigue, often accompanied by diarrhea, sore throat, runny nose, muscle pain, and so on. Severe cases can rapidly develop into respiratory distress syndrome, septic shock, metabolic acidosis and coagulopathy, and multiple organ failure [1]. SARS-CoV-2 is a coronavirus with a membranous envelope, in a spherical or oval shape, often polymorphic, with a diameter of 60–140 nm [1]. The viral infection involves entry of SARS-CoV-2 virus into host cells [2–5] mainly via a cell receptor angiotensin-converting Enzyme II (ACE2). COVID-19 can be transmitted through human saliva and physical contact, with an incubation period of 1–14 days. It is also highly contagious during the asymptomatic period, with 44 percent of transmission occurring before the onset of symptoms. The viral load in the patient's saliva peaked during the first week of the onset of new COVID-19 symptoms such as fever and cough [3, 6–9]. No precise vaccine or drug has been approved to prevent COVID-19 effectively, leading to the rapid global spike of confirmed cases. Until 3rd August in 2020, more than 17 million cases of COVID-19 infections and with more than 680,000 deaths have been confirmed in 209 countries and regions worldwide.

In China, anti-viral drugs such as interferon-α, ribavirin, Lopinavir/Ritonavir, chloroquine phosphate and arbidol, as well as a combination with traditional Chinese medicine (TCM) are recommended to treat COVID-19 [1]. However, Lopinavir or Ritonavir did not outperform the benefits to those of the standard treatment in hospitalized adults with severe COVID-19 [10]. On the other hand, TCM has been observed to play an important role in preventing and controlling the epidemic. According to data released by Chinese Center for Disease Control and Prevention on 23rd March 2020, 74,187 of the newly diagnosed patients of COVID-19 in China were treated with Chinese medicine, accounting for 91.5 percent of total patient population at that time. In Hubei Province, 61,449 patients were treated with TCM, accounting for 90.6 percent [11]. Clinical observation showed that the total effective rate of Chinese medicine reached more than 90% [11]. TCM efficiently relieved the symptoms of the patients and promoted a fast recovery of their bodies, thus effectively reducing the number of patients whom changed from mild to severe conditions, as well as the overall mortality rate of the patients [11].

At present, a large amount of research work has been invested in searching for the treatments of COIVD-19 worldwide. In China, promising therapies for COIVD-19 have been recommended according to the accumulated experiences of TCM in fighting with various epidemics during the long history. In this paper, we mainly focus on summarizing the advantages of TCM in treating COVID-19 and several advanced techniques and concepts for drug screening of anti-COVID-19 drug candidates. Varieties of in vitro and in vivo models for drug screening are also introduced. We expect that this review to provide researchers valuable ideas and approaches in fast finding out the precise therapies and/or effective drugs against COVID-19.

## COVID-19

As a single-stranded RNA virus belonging to the C family of coronavirus, SARS-CoV-2 features four structural proteins, S protein, N protein, M protein, and E protein [12]. The current results suggest that the virus enters cells by binding to the host surface receptor, ACE2, via the S protein, and then fuse with the host cell [13]. Compared with SARS-CoV and MERS-CoV, the pathogenicity of SARS-CoV-2 is lower but is easier to spread [14]. Whether fecal–oral route transmission of SARS-CoV-2 plays an important role in spreading the virus, reminiscent of SARS-CoV, needs to be further studied for confirmation. Of particular concern is whether SARS-CoV-2 will become as seasonal as community-acquired HCoV. The heredity, pathogenicity, and continuous transmission of SARS-CoV-2 in the human body will influence the developing trend of COVID-19 which is currently outbreaking globally [14].

To the origin of SARS-CoV-2 virus, researchers have thus far put forward two hypotheses: (1) It is a natural selection in animal hosts before zoonosis transfer; (2) It is natural selection after the zoonosis shift [15]. Both hypotheses are helpful for further investigate and elucidate how the virus initially transmitted from animals to humans. At this stage, some researchers believed that the virus was originally from a natural host of bat, a report show that the 2019-nCoV is 96% identical at the whole-genome level to a bat coronavirus [3]. The RTG13 sequence helped reveal key RBD (receptor-binding domain) mutations and multiple base-cutting sites. The virus may recombine to evolve in wild animals such as pangolins, and then cross the species barrier to humans through other intermediate hosts [16]. As of April 5, SARS-CoV-2 has been observed and reported to be effectively replicated in ferrets and cats, and transmitted through respiratory droplets in cats [17].

At present, there are two main detection methods for SARS-CoV-2 virus: nucleic acid detection and immunological detection. Nucleic acid detection methods include gene sequencing, fluorescent quantitative PCR, digital micro-drop PCR, gene chip, and loop-mediated isothermal amplification. Immunoassays include immunochromatographic strips, Elisa, and chemiluminescence immunoassay [12]. Since the outbreak of COVID-19 pneumonia, there has been no precise drugs for treatment of this disease with regulatory approval. Next, we will review and make a comprehensive analysis of drug targets, as well as possible therapeutic paradigms such as vaccine and therapeutic antibody, general antiviral drugs, protease inhibitor, and convalescent plasma therapy.

The S protein is the receptor via which the virus binds to the host cell, and participates in cell fusion. It is a key target for the development of antibodies, vaccines (especially polypeptide and mRNA vaccines), small molecular compounds against this virus. The receptor for SARS-CoV-2 infected cells is ACE2, which is similar to SARS-CoV and also relies on transmembrane serine protease, is also a target for drug development [18]. According to the previous research of SARS-CoV, a RBD region on the correct conformation of S protein may be an ideal immunogen for vaccine design and development [16]. Besides, an inhibitor of lipoprotein nucleotide fusion based on HR2 sequence was designed, called IPB02, which is highly active in SARS-CoV-2 S protein-mediated cell-to-cell fusion and pseudovirus infection [19]. SARS-CoV-2 major protease (Mpro) is a key CoV enzyme, which plays a pivotal role in mediating viral replication and transcription, making it an attractive drug target. Shanghai Institute of Materia Medica, in collaboration with several institutions, has designed and synthesized two lead compounds against SARS-CoV-2 Mpro, both of which have shown effective anti-infectious activity [19]. Besides, activation of NLRP3 (nucleotide-binding domain, leucine-rich-containing family, pyrin domain-containing-3) inflammatory bodies is lacking in bats, so the use of NLRP3 inhibitor MCC950 may be helpful in the treatment of COVID-19 [14]. Viral 3-chymotrypsin-like cysteine protease (3CLpro), which controls the replication of coronavirus, is a drug target of SARS-CoV and MERS-CoV. Nine potential anti-SARS-CoV-2 active compounds have been identified via this molecular pathway [20].

As the most effective medical means of epidemic prevention and control, vaccine can effectively block the spread of the virus [21]. At present, China is focusing on the simultaneous implementation of three technical routes: influenza vector vaccine, recombinant protein vaccine, and nucleic acid vaccine. Led by academician Wei Chen from the Medical Research Institute of the Academy of Military Medical Sciences, the team has successfully developed a recombinant new crown vaccine, which is now in Phase I clinical trial (registration number of recombinant SARS-COV-2 vaccine (adenovirus vector) registration No. ChiCTR200030906). At the same time, some international pharmaceutical companies include Gene One Life Science Inc. and Inovio Pharmaceuticals Inc. have conducted relevant animal experiments to develop the COVID-19 vaccine. Moderna and the Vaccine Research Center of the National Institute of Allergy and Infectious Diseases conducted the first phase I clinical trial of a novel lipid nanoparticle (LNP)-encapsulated mRNA-based vaccine, mRNA-1273, which encodes the spike protein (S protein) of SARS-CoV-2, began in the United States [22, 23].

Scholars proposed two very far-sighted global solutions in the event of a new virus that triggers a pandemic in the future. One solution is to establish a global distribution of production capacity, where surveillance in animal reservoirs combined with characterization of the virus can identify members of the virus family that are likely to cause a pandemic, candidate vaccines using these isolates can then be produced, and this capability can be activated if new viruses emerge. This strategy is currently applied to H5 and H7 avian influenza vaccines. Another solution is the development of a broad-based protective vaccine covering the entire family or genus of the virus, which is currently underway for influenza viruses and may be applied to coronaviruses in the future [24].

Therapeutic antibodies are also specific therapeutic drugs. SARS-COV-2 and RBD of SARS-CoV have high homology. The strong neutralizing antibody CR3022, which targets SARS-CoV RBD, can be effectively combined with SARS-CoV-2 RBD. Thus it is a potential anti-SARS-CoV-2 antibody [18]. Mode RNA Therapeutics, Wuxi Biologics and Vir Biotechnology are developing monoclonal antibody for SARS-CoV-2 and several pre-clinical studies have been completed and clinical trials have also begun.

Vaccines, in the form of monoclonal antibody, oligopeptides, and peptide molecules, take months to years to develop. Considering approved antiviral drugs against a variety of viruses such as HIV, HBV, HCV, SARS, and MERS, Guangdi Li et al. recommended some of these current antiviral drugs for treating COVID-19, such as nucleoside inhibitors Favipiravir, Ribavirin or Ridgisivir [25]; common antiviral drugs Chloroquine phosphate, Favipiravir, and Arbidol; protease inhibitors disulfiram or Lopinavir/ritonavir and immunosuppressants may also work against COVID-19 [25]. In the absence of specific therapeutic drugs, convalescent plasma therapy (CPT), a passive immunotherapy, has been one of the potential treatment options for COVID-19 [26]. The strategy has been used to treat flu, viral infections, SARS, and other infectious diseases. Since the plasma who meets the medical requirement is rather limited, and the quality of blood products is demanding, the use of CPT has failed to popularize, in spite of demonstrated efficacy [26].

Therefore, researches on exploring and developing effective drugs for preventing and treating COVID-19 are necessary and urgent. As very limited modern medicine has shown clinically approven efficacy for COVID-19, lessons can be drawn from the effective treatment experience from the use of the combination of TCM and Western medicines. During the screening of effective drugs and potential formulations or medicinal combinations, a variety of in vitro and in vivo screening models has been employed. Therefore, disease models and some core technologies would be introduced in the following paragraphs for facilitating researchers to screen drugs for treating COVID-19.

## Experimental models

### Cell Models

Using in vitro models (e.g. certain cell lines), the anti-viral effect of a drug candidate could be quickly predicted preliminarily. At present, the most commonly used cell line in antiviral test of Chinese medicine is MDCK (Madin-Darby Canine Kidney) cells. In addition, Hep-2, A549, MBCK, and mouse macrophage Raw264.7 are also frequently applied for screening of antiviral drugs.

#### MDCK cells

MDCK cells are susceptible to multiple influenza virus strains, supporting numerous passages of virus replication (capable of 35 consecutive rounds), and are widely used in antiviral activity assessment, mechanism research, and influenza vaccine development. MDCK cells are the most widely used cell lines for antiviral research in TCM [27–29]. Single herb such as *Scutellariae Radix* (黄芩) [30], *Isatidis Radix* (板蓝根) [31], *Lonicerae Japonicae Flos* (金银花) [32], *Houttuyniae Herba* (鱼腥草) [32], etc. and TCM formulations such as Fufangyizhihao granule (复方一枝蒿颗粒) [33] and Lianhua Qingwen capsule (连花清瘟胶囊) [33] have shown potentials on attenuating the proliferation of a/PR/8/34(H1n1), a/Aichi/2/1968(H3n2) and other influenza viruses in MDCK cells.

#### A549 cells

A549 human lung adenocarcinoma epithelial cell line is a commonly used tool to investigate the interactions between virus and host, the biochemistry of viral proteins, and to analysis genes that are overexpressed to suppress viral infections, to perform large-scale drug screening, for example, screening for CRISPR activation, [34]. It was previously demonstrated that TCM *Reseda odorata* (木犀草) decreased the Caspase-9, Caspase-8 and Caspase-3 expression in A549 cells infected with H1N1 virus [35] and *Chuanxiong Rhizoma* (川芎) [36]. In addition, the inhibitory effects of *Scutellariae Radix* on a 1/FM/166/85(H1N1) virus and Influenza A and B viruses have been demonstrated in A549 cell line [37, 38].

#### Vero E6 cells

Researchers have used Vero E6 cells to understand COVID-19. For instance, Vero E6 cells were used to test the antiviral activity of Lianhua Qingwen capsule against SARS-COV-2 [39]. More interestingly, recent studies on Vero E6 cells demonstrated that TMPRSS2 could enhance SARS-CoV-2 infection, which was in common with Middle East respiratory syndrome and SARS-CoV. Given the various variants containing point mutations or 15–30-bp deletions (Del-mut) found accordingly at the S1/S2 junction via plaque purification of Vero-E6 cells cultured with SARS-CoV-2 and the fact that adaptive function could disappear due to replication of permissive Vero-E6 cells, it was suggested that strong selective pressure might be responsible for SARS-CoV-2 infection in humans promoted by the unique cleavage motif [40]. Additionally, a TMPRSS2-expressing VeroE6 cell line was considered helpful for propagating and isolating SARS-CoV-2, because it was easily accessible to infection of SARS-CoV-2 [41].

#### Other cells

Yi Zhi Hao Granule has been reported to present obvious anti-H1N1 activity on human laryngeal Carcinoma epithelial cell Hep-2, and can alleviate lung injury and reduce the mortality of infected mice [42]. Xiaoqinglong decoction (小青龙汤) can effectively inhibit the infection of Hep-2 cells by the human respiratory syncytial virus [43]. In addition, mouse macrophages, Raw264.7 are increasingly favored for screening antiviral drugs. For instance, Yinhua Ping Gan Granule (银花平感颗粒) was observed to efficiently inhibit the proliferation of a/PR/8/34(H1N1) virus in Raw264.7 cells, which might be related to the following elements: regulates type I interferon and pattern recognition receptor signaling pathway; up-regulates the expression of IFN-γ and anti-myxvirus Protein 1; down-regulates the expression of IL-6, TNF-α and phosphorylated TANK binding kinase 1, and signal transduction and transcription activator 1 [33].

#### Animal models

As in vitro screening can only offer potential drugs against COVID-19, proper animal models have to be established to further understand COVID-19 disease evolvement and to screen drugs for effective treatment, before possible clinical trials. Researchers are testing mice, rats, ferrets, and even monkeys to answer key questions about the diseases and to fast-track potential drugs and vaccines for clinical trials.

After outbreak of COVID-19, China Institute of Laboratory Animal Sciences (CNILAS) has taken the lead in establishing the disease models in transgenic mice and rhesus monkeys with COVID-19, enriching the understanding of the etiology and pathology of COVID-19. With the model, 5 patent medicines have been thoroughly evaluated in vivo for potential treatment of COVID-19, and 6 vaccines and 4 more patent medicines are currently evaluated [44]. It was reported that Academician Dr. Zhong Nanshan's team has created the world's first non-genetically modified mouse model infected with SARS-COV-2. Compared with the traditional receptor transgenic mouse model, this model has a shorter construction time and does not need to reproduce; therefore, it is suitable for large-scale popularization in a short time. This model is useful for in vivo validation of antiviral drug and protective neutralizing antibodies, and vaccines. Besides, the model can be used to study the immune response and pathogenesis of SARS-COV-2 in vivo. In addition to mice and rhesus monkeys, other animals similar to human viral infections have been used in previous studies to screen for an antiviral drug.

#### Mouse

Mouse plays an important role in researches on the pathogenesis of respiratory diseases associated with human virus infection [45–49]. Some researchers are currently using mice as an animal model to test drugs and vaccines and to investigate the nature of the infection of SARS-CoV-2 [49–51]. However, mice often shrug off infection with SARS-CoV-2 and only exhibited a relatively mild clinical disease [24, 52], because mouse ACE2 receptor features several differences from that of humans [53]. For instance, out of 29 key amino acids in this important domain,11 in mouse were different from that in human [54]. Luckily, engineering mice that could express both the human and mouse version of the receptor’s gene, ACE2, has been suggested as an effective way to remove the roadblock. In fact, in a study led by Qin Chuan on SARS, engineered mice that could express human ACE2 protein was successfully established, leading this Chinese team pioneered the establishment of a SARS-CoV-2 infected hACE2 transgenic mouse model [54]. During investigation of the pathogenicity of the virus in this mouse model, weight loss and virus replication in the lung was observed. Therefore, this model still needs to be optimized since the weight loss and signs of pneumonia were mild and very different from that of humans [54]. Currently, researchers are accelerating animal model research for SARS-CoV-2, and the transgenic mice developed for SARS-CoV are in short supply. For example, an effective and convenient novel mouse model in evaluating in vivo protective capacity of the SARS-CoV-2 vaccines was developed through stitching the human gene for ACE2 into an adenovirus by Perlman et al. in his unpublished work, in which mice was infected with it then receptor was created by some of their lung cells [55]. Surprisingly, 20% of weight loss, which was greater than twice in Qin’s study, ruffled fur, other illness sign with no death were occurred in those mice infected with SARS-CoV-2. In addition, pacific inflammatory responses and pneumonia were found in the most recent study, in which both young and aged wild-type BALB/c mice were effectively infected with mouse-adapted SARS-CoV-2 at passage 6 (MACSp6) [55]. Furtherly, a receptor-binding domain (RBD)-based SARS-CoV-2 subunit vaccine was found to conferred full protection against SARS-CoV-2 MACSp6 challenge and elicited highly potent neutralizing antibodies, and the protective activity of it was determined in vivo.

#### Rat

Of the 29 amino acids in the key region of the human ACE2 receptor that binds to the virus, 13 amino acids differ from that of the rat [54]. Therefore, rats might not be a suitable model for studying SARS-CoV-2 infection. In terms of susceptibility to SARS-CoV-2, rats also had no advantage. However, the large size of rats has some advantages in many experiments that require repeated blood collection**.**

#### Hamster

Syrian hamsters are gaining increasing attention in the fight against COVID-19. In hamster, only 4 of 29 amino acids of ACE2 receptor are different from those of human [56]. Despite of the subtle symptoms, coronavirus was found to easily infect hamsters and cause serious acute respiratory syndrome (SARS) as early as fifteen years ago, nevertheless, hamster did not catch much attention as an effective animal model for the disease at that time [57]. Now, however, the tables have turned in the case of COVID-19 since this model comes under spotlight again caused by a related virus, SARS-COV-2. The first new hamster model was published in January by a physician-scientist of the University of Hong Kong (HKU) Jasper Fuk-Woo Chan, whose work become one of the earliest studies in which asymptomatic infection and human-to-human transmission of SARS-COV-2 were reported. Eight hamsters were infected with SARS-CoV-2 by Chan and his co-workers in their study, afterwards, a body weight loss, ruffled fur, lethargy, a hunched posture and rapid breath occurred with high levels of SARS-COV-2 found in the lungs and intestines, tissues studded with the virus’ target, ACE2 of the animals [58]. Interestingly, another team also at HKU. Led by Hui-Ling Yen suggested comparable results closely behind of this study [58]. What’s more inspiring, those hamster studies may contribute to understand the spreading way of the virus. In both above studies, transmission of SARS-COV-2 happened whenever an uninfected hamster was put together with an infected one in a cage, apart from the possible transmission through respiratory droplets, Chan and his colleagues also noticed the fact that hamsters ate feces, and therefore, a fecal–oral spread way could not be ruled out [58].

#### Ferret

Ferret shares remarkably similar lung physiology with humans, unsurprisingly, it is widely used in researches of respiratory viruses that infected humans and as one of the best animal models for another respiratory disease and influenza [59]. They are susceptible to human influenza viruses and various of the clinical symptoms of influenza infection in humans can be found on them [59]. A team of researchers in China recently found that SARS-COV-2 could replicate in the upper respiratory tract of ferrets for up to eight days, without causing severe disease or death. The authors believed that the ferret could be a potential candidate animal model for assessing vaccine candidates and antiviral drugs against COVID-19 [60]. By contrast, in the same study, livestock including chickens, pigs and ducks were demonstrated to not susceptible to SARS-COV-2, while dogs had low susceptibility and cats were highly susceptible to the virus. Besides, Kim et al. [61] reported similar results. They found that ferrets were highly susceptible to SARS-COV-2 infection, and exhibited the elevated body temperature and virus replication. Although fatalities were not observed, ferrets effectively transmitted the virus by direct or indirect contact, recapitulating human infection and transmission. More importantly, Kim also suggested that older ferrets might serve as a better animal model than younger ones. Regardless of the unclear reasons, it is widely accepted that SARS-COV-2 strikes the elderly much harder than the younger in humans, and similar phenomenon happened in the ferrets. Kim had observed more severe symptoms causing loss of white blood cells and platelets in older ferrets infected with the virus, besides, 93% of older ferrets died of viral infection while no symptoms showed in younger ones infected with the same virus [62], which indicated again that ferrets might be an ideal animal model for understanding and defeating against COVID-19 since it presents similar features.

#### Primate

Non-human primates, especially rhesus monkeys, have similar immune responses to human viral infections due to their genetic and physiological similarities [63]. Non-human primates are the closest species to human being who is subject to the infection of viruses [64]. Different species of monkey have been infected with SARS-COV-2 by intense efforts that closely followed by the isolation of the virus from human. A team from Wuhan Institute of Virology, Chinese Academy of Sciences, published the first study of SARS-COV-2 infected rhesus monkey model [65]. They found that SARS-CoV-2 caused acute localized-to-wide to spread pneumonia as proved by pathological studies in Rhesus Macaques, although without obvious clinical symptoms of respiratory disease.

Another research team observed more serious interstitial pneumonia in old monkeys infected by SARS-COV-2 than that in young monkeys, which provided insights into the pathogenic mechanism and may facilitate the development of vaccines and therapeutics against SARS-COV-2 infection. In their study, two 15 years old and three 3–5 years old rhesus macaques were infected with SARS-COV-2, then viral replication of anal swabs, nasopharyngeal swabs and the lung in old monkeys was found to be more active than that in young monkeys 14 days after the virus infection [44]. Typical interstitial pneumonia characterized by thickened alveolar septum along with edema and inflammation was developed in monkeys, and diffuse severe interstitial pneumonia was observed in old monkeys. The research team led by Qin Chuan has obtained similar conclusions. They further found that SARS-COV-2 had a conjunctival infection transmission route in the rhesus monkey model, which suggested conjunctival infection could cause mild COVID-19 symptoms [66]. Another critical finding was that the reinfection by SARS-COV-2 of rhesus macaques might be decreased by a remarkably enhanced neutralizing antibody response, suggesting that primary SARS-COV-2 infection might contribute to protection of monkeys from subsequent reinfection [67].

Besides, some scientists inoculated cynomolgus macaques with SARS-COV-2 or MERS-CoV and compared with historical SARS-CoV infections and found that the severity of SARS-COV-2 infection was intermediate between that of SARS-CoV and MERS-CoV [68]. Lu et.al recommended that *Macaca mulatta* could be used to investigate viral pathogenesis and evaluate vaccines and drugs of COVID-19. Two different families of non-human primates, including New World monkeys (6 *Callithrix jacchus*) and Old World monkeys (6 *Macaca fascicularis,* 12 *Macaca mulatta*) were involved in their study, and they reported that *Macaca mulatta* was most susceptible to SARS-CoV2 infection, followed by *Macaca fascicularis* and *Callithrix jacchus* [69].

A MERS-CoV primate model was successfully established in a latest study, which firstly reported the dose-dependent effects of highly pathogenic coronavirus infection of primates and used a route of infection (small particle aerosol) with potential relevance to MERS-CoV transmission in humans with twelve African green monkeys [70]. African green monkeys may serve as primate model for COVID-19, with considerably potential value in viral pathogenesis and therapeutic development researches [70]. Taken together, monkeys might be used as suitable animal models to evaluate vaccines and drugs for COVID-19.

## Feasible scheme and core technology group of prescription drug screening

### Drug screening based on data mining

Through data mining to screen the clinical use of the TCM Formula for COVID-19, high frequency/core drugs in TCM Formula could be selected. Also, the induction and analysis of four properties and five tastes (四气五味) and meridian tropism (归经) of many Chinese medicines from TCM Formula could be made. Effective guidance for the prevention as well as clinical treatment of COVID-19 could be provided through investigations of the characteristics of commonly used medicine.

Through data mining, the key Chinese medicine to prevent new coronary pneumonia is *Saposhnikoviae Radix* (防风), *Atractylodis Macrocephalae Rhizoma* (白术), *Astragali Radix* (黄芪), *Glycyrrhizae Radix et Rhizoma* (甘草), etc.[71]. Based on the basic knowledge of TCM and clinical experience, three drug combinations were rendered: a) *Saposhnikoviae Radix*, *Atractylodis Macrocephalae Rhizoma*, *Astragali Radix*, *Glycyrrhizae Radix et Rhizoma*, *Platycodonis Radix* (桔梗), *Lonicerae Japonicae Flos*, *Forsythiae Fructus* (连翘), *Citri Reticulatae Pericarpium* (陈皮), *Eupatorii Herba* (佩兰), *Lilii Bulbus* (百合); b) *Dryopteridis Crassirhizomatis Rhizoma* (贯众), *Atractylodis Rhizoma* (苍术), *Pogostemonis Herba* (广藿香), *Coicis Semen* (薏苡仁); c) *Mori Folium* (桑叶), *Chrysanthemi Flos* (菊花), *Ophiopogonis Radix* (麦冬), and *Adenophorae Radix* (南沙参). By analyzing 96 effective prescriptions, the four properties (四气) were mainly concentrated in Warm (温), Cold (寒) and Ping (平), the five tastes(五味) were mainly in Bitter (苦), Spicy (辛) and Gan (甘), and meridian tropism were mainly in lung, stomach, and spleen. The high-frequency herbs are *Glycyrrhizae Radix et Rhizoma*, *Armeniacae Semen Amarum* (苦杏仁), *Gypsum Fibrosum* (生石膏), etc. The high-frequency herb combinations are *Gypsum Fibrosum-Armeniacae Semen Amarum*, *Gypsum Fibrosum- Glycyrrhizae Radix et Rhizoma*, *Glycyrrhizae Radix et Rhizoma-Armeniacae Semen Amarum*, etc. Core combination is *Descurainiae Semen- Armeniacae Semen Amarum- Gypsum Fibrosum*, *Pogostemonis Herba- Zingiberis Rhizoma Recens- Magnoliae Officinalis Cortex* (广藿香-生姜-厚朴), *Ophiopogonis* Radix- *Armeniacae Semen Amarum- Scutellariae Radix* and so on. The integral principle of treatment is supporting the body and dispelling pathogenic factors, taking into account both Qi and Yin. Through the analysis of 212 Chinese medicine prescriptions in various regions of China, the top 4 herbs used most frequently were *Glycyrrhizae Radix et Rhizoma*, *Armeniacae Semen Amarum*, *Ephedrae Herba* (麻黄), and *Pogostemonis Herba* [72]. From the meridian tropism point of view, the Lung Meridian, Stomach Meridian are dominant. According to the classification of efficacy, the top four herbs used most frequently were heat-clearing herbs, antipyretic herbs, qi-invigorating herbs, and yin-tonifying herbs. It was found that in the prescriptions of TCM for the prevention and treatment of COVID-19 in various regions, the main methods were to clear away heat and reinforce deficiency, and to strengthen the spleen and stomach. In the later period, most of TCM used were tonifying Qi and Yin.

### Drug screening based on molecular docking technology

Molecular docking is a model based on applied mathematics, biology, and computers to predict the binding affinity of small molecules to specific receptors, which can be used to predict drug affinity at target binding sites and drug-specific metabolic enzyme interactions to better understand the complexity of living systems.

Studies have shown that SARS-CoV virus infections are through the expression of S-protein and human ACE-2 binding, which leads the virus to enter the cells [65, 73]. Through molecular docking technology, 46 active components from TCM formula with high binding capacity could act on the binding region of SARS-COV-2 S-protein of human ACE2 protein. The screened components mainly belong to *Lonicerae Japonicae Flos*, *Mori Folium* and other seven herbs [74]. Papain-like protease (PLP) plays an important role in the replication of coronavirus. In the process of virus replication, the small RNA virus first encodes a large polymer precursor protein, and then the polymer protein is hydrolyzed to produce a functional protein, the hydrolysis process is mainly completed by 3CLpro. Therefore, it is important to search for inhibitors of 3CL hydrolase of SARS-COV-2 coronavirus for the prevention and treatment of COVID-19.

Through molecular docking technology, *Trichosanthis Fructus* (瓜蒌) and *Fritillariae Cirrhosae Bulbus* (川贝母), obtained by PLP inhibitors screening and ACE2 inhibitors screening respectively, were parts of the Sangbei Zhisou Powder (桑贝止嗽散) and Xiaoxianxiong Decoction (小陷胸汤), which had the effect of clearing away heat and phlegm [75]. They can be applied to the syndrome of cough with slight dyspnea caused by external pathogenic factors in the early stage of viral action*. Radix et Rhizoma Rhei* (大黄) and *Trichosanthis Fructus*, screened by Mpro and PLP respectively, are in Maxing Shigan Decoction (麻杏石甘汤) and Xuanbai Chengqi Decoction (宣白承气汤). They can be applied to syndrome types of the lung obstructed by pathogenic heat or blocked by toxin, and viscera-solid knot in the severe stage of the disease. *Mori Folium, Lonicerae Japonicae Flos and Forsythiae Fructus* obtained by ACE2 inhibitors screening have been included in the Sangju Beverage (桑菊饮) and Yinqiao Powder (银翘散), which can be used in the early stage of the disease, such as the syndromes of warming evil invading the lung and slight cough and asthma.

### Network Pharmacology

Network pharmacology, which constructs network models centering on medicinal materials and disease targets, can provide a reasonable experimental basis and a brand-new research idea for discovery of new drugs and excavation of potential drugs. Combining the study of the entire network with the holistic characteristics of TCM, network pharmacology can be used to study the basic theory of TCM from the perspectives of multi-approach, multi-component, and multi-target. Screening the active substances, predicting the target protein, finding the signal pathway, constructing the network and analyzing it by network pharmacology, so the potential mechanism between the active components, target proteins and the network of pathways related to the occurrence and development of diseases may get understood, consistent with the overall concept of TCM. Subsequently, the function of the key active components and COVID-19 related proteins can be studied by molecular docking technique, and the potential material basis of treating COVID-19 with prescription can be therefore explored, which could provide theoretical reference for preventing and treating COVID-19 with prescriptions.

Using Jean-Baptiste de Lamarck genetic algorithm and virtual docking technology, small molecular components of Chinese herbal medicine were screened from the component library of Chinese herbal medicine based on S-protein and ACE2. Using S-protein as the target, 12 components were screened out, including in *Artemisia Annua Herba* (青蒿), *Salviae Miltiorrhizae Radix et Rhizoma* (丹参), *Scutellariae Radix*, *Pinelliae Rhizoma* (半夏), *Glycyrrhizae Radix et Rhizoma*, *Bupleuri Radix* (柴胡) and so on. Using ACE2 as the target, 77 components were screened, including in *Salviae Miltiorrhizae Radix et Rhizoma*, *Glycyrrhizae Radix et Rhizoma*, *Scutellariae Radix*, *Ephedrae Herba*, *Armeniacae Semen Amarum*, *Bupleuri Radix*, etc. Finally, potential inhibitors of carnofone and dihydrodanshen lactone were identified [76]. Through the analysis of Weixuening mixture by network pharmacology, 326 components and 555 related targets were obtained, of which 35 targets corresponding to 101 components were closely related to COVID-19. More specifically, herbs-active compounds-targets network and Gene Ontology function enrichment analysis as well as Kyoto Encyclopedia of Genes and Genomes pathway enrichment analysis were performed by Cytoscape software and Database for Annotation, Visualization and Integrated Discovery. The enrichment analysis of the target pathway showed that Weixuening mixture mainly acted on infectious disease, inflammatory reaction, and immune regulation. It is suggested that Weixuening mixture may play a potential role in the prevention and treatment of COVID-19 by regulating the immune and inflammatory responses [77]. Besides, with network pharmacology, it was found that the core active compounds in Yupingfeng powder (玉屏风散) regulates multiple signal pathways by binding with ACE2 to ESR1, AR, PTGS2, and other targets to prevent and treat COVID-19 [78]. Jinhua Qing Gan Granule (金花清感颗粒) can regulate the signal pathway of PTGS2, HSP90AB1, HSP90AA1, PTGS1, and NCOA2 by combining 2019-nCoV3CL hydrolase and ACE2 to prevent and treat COVID-19 [79].

Taking advantage of the nascent computer technology and professional software, researchers could quickly screen out the regular pattern of anti-COVID-19 drugs according to the existing pharmacological knowledge. In combination with clinical experience, it can further provide effective suggestions to clinical medication. This method can also screen out the active substances, target proteins, and possible signal pathways of related drugs. The application of computer virtual screening technology can greatly reduce the blindness in the screening of prescriptions and medicines, and save manpower, material, and financial resources. However, this method is limited by the information of drug structures and targets.

### Biochips

Biochip technology can detect numerous information simultaneously, which is helpful to explore the molecular mechanism of compounds from Chinese medicine, optimize the dosage and prescription. Gene chip can connect the characteristics of multi-component, multi-target, multi-pathway action, and gene expression together; compare their difference in expression profiles; and determine the corresponding gene expression targets. According to the expression of organ specificity and the level of expression and the compound theory of monarch, minister, assistant and guide, and correlates with medication dose. At the same time, according to the interaction of the corresponding gene targets of different compatibility, the close relationship between each compound medicine can be analyzed, and the material basis of drug action and internal compatibility law would be elucidated. Through gene chip technology observing the changes of gene transcription in an apoptotic signal pathway, a study found that Luteolin could down-regulate the differentially expressed genes of CASP3, CASP8, and MyD88, and interfere with cell apoptosis in A549 cell infected by H1N1 [80]. Therefore, biochip technology presents great potential being used for screening TCM formulas in treating COVID-19.

## Conclusions and perspectives

The response of clinical studies of TCM in the prevention and treatment of COVID-19 have been very rapid, and the current registration scheme covers the whole process of disease prevention, treatment, and rehabilitation. TCM formula has the characteristics of multi-component, multi-target, and multi-way synergy, which are the advantages of clinical treatment of TCM. However, perspective from the modern medicine, clarification of the relationship between chemical basis and biological mechanisms for TCM is still a challenge to be concerned. The substance basis, curative effect, and mechanism of TCM formula need to be further investigated and demonstrated. Besides, many means of TCM diagnosis and treatment rely on personal experience. To guide the clinical medication, it is important to establish an objective and standardized diagnosis and treatment standards. Traditional methods for screening antiviral Chinese medicines are limited by the experimental cycle; since the high infectivity of SARS-COV-2, the high requirements of laboratory conditions greatly lag the progress of drug screening.

The common techniques of Computer-Aided Drug Design (CADD) include molecular docking, reverse docking, QSAR, pharmacophore, and prediction of drug absorption, distribution, and metabolic transport. Discovery and optimization of drug targets and the application of lead compound in the absorption, distribution, metabolism, excretion, and toxicity prediction of compounds have greatly accelerated the process of drug screening. CADD is also a tool for studying TCM, expounding the mechanism of action of TCM, and optimizing the development of new TCM prescriptions. Among them, the technologies of molecular docking (DOCK), inverse molecular docking (INVDOCK), similarity search, substructure search, pharmacophore search, pharmacophore screening, and chemical component toxicity prediction are of great significance to the study of TCM and its prescription. It is an important tool to explain the mechanism of TCM scientifically and reasonably [80]. In order to find out the potential Chinese medicines or active ingredients against COVID-19, scholars used data mining and network to mine the high-frequency Chinese medicines and formulas from ancient prescriptions, then explored binding rates between the main ingredients in high frequency Chinese medicines and the key targets of SARS-COV-2 via using molecular docking approach. Finally, a network pharmacology was used to uncover the potential molecular mechanism [81]. Researchers can quickly screen out potential medicines by CADD, but further animal experiments and clinical studies should be performed to confirm the safety and effectiveness of these medicines.

The TCM prescription is composed of different compounds, and its function is to target multiple target genes. It can directly inhibit viruses by target protein, and indirectly inhibit virus by human target protein. Therefore, predicting the gene-target interactions of active components could help to decipher the mechanism of multi-target action for TCM. In the search for new antiviral drugs, studies have been conducted on molecular docking and molecular dynamics simulations to identify components or derivatives of Chinese medicines or TCM formulations that can block channel activity or drugs at sialic acid binding sites. A study examined six viral proteins that could be targeted by antiviral drugs, including neuraminidase, Hemagglutinin, matrix protein 1, M2 proton channel, nucleoprotein, and nonstructural protein 1 [82]. Molecular docking techniques were used to identify potential inhibitors of 13,144 TCM compounds. The results showed that 56 compounds could inhibit more than two drug targets simultaneously. The interactions of these compounds with host targets were studied by molecular reverse docking. Finally, it was found that 22 compounds inhibitors could stably bind to host targets with high binding free energy [82].

Significant research works have provided indispensable insights into the technology and methodologies in screening drugs treated COIVD-19 that likely contribute to the development and progression of interdisciplinary strategies in the understanding to prevent and treat COIVD-19. As stated prior, in vivo and in vitro models have been developed to verify the effectiveness of Anti-COVID-19 drugs. But there is still wanting in the most commonly used model that provides an inexpensive, simple and reproducible model to study COIVD-19. It is our hope that our further understanding of the role of frontier concepts and core technologies in screening Anti-COVID-19 drugs that may lead to new therapeutic targets for preventing and treating COVID-19.

## Data Availability

Not applicable.

## Abbreviations

ACE2: Angiotensin converting enzyme 2
CADD: Computer-aided drug design
CASP3: Caspase 3
CASP8: Caspase 8
CNILAS: China Institute of Laboratory Animal Sciences
COVID-19: Coronavirus Disease 2019
CPT: Convalescent plasma therapy
CRISPR: Clustered Regularly Interspaced Short Palindromic Repeats
DOCK: Molecular docking
ESR1: Estrogen receptor 1
HBV: Hepatitis B virus
HCV: Hepatitis C virus
HIV: Human immunodeficiency virus
HR2: Heptad repeat 2
HSP90AA1: Heat Shock Protein 90 kDa Alpha A1
HSP90AB1: Heat Shock Protein 90 kDa Alpha (cytosolic), Class B Member 1
H1N1: Hemagglutinin 1 Neuraminidase 1
IL-6: Interleukin-6
INF-γ: Interferon-γ
INVDOCK: Inverse molecular docking
MACSp6: Mouse-adapted SARS-CoV-2 at passage 6
MDCK: Madin-Darby Canine kidney cell
MERS-CoV: Middle East Respiratory Syndrome Coronavirus
Mpro: Major protease
MyD88: Myeloid differentiation factor 88
NCOA2: Nuclear receptor coactivator 2
NLRP3: Nucleotide-binding domain, leucine-rich-containing family, pyrin domain-containing-3
PCR: Polymerase chain reaction
PLP: Papain-like protease
PTGS1: Prostaglandin endoperoxide synthase 1
PTGS2: Prostaglandin-endoperoxide synthase 2
RBD: Receptor-binding domain
SARS: Severe acute respiratory syndrome
SARS-CoV: Severe acute respiratory syndrome coronavirus
SARS-CoV-2: Severe acute respiratory syndrome coronavirus 2
TCM: Traditional Chinese Medicine
TMPRSS2: Transmembrane protease serines 2
TNF-α: Tumor necrosis factor-α
